# Dietary Vitamin K Intake and HPV-Infection Status Among American Women: A Secondary Analysis From National Health and Nutrition Examination Survey Data From 2003 to 2016

**DOI:** 10.3389/ijph.2022.1604616

**Published:** 2022-08-24

**Authors:** Yinhui Jiang, Shu Xu, Jinzhi Lan, Jinjuan Zhang, Tengxiang Chen

**Affiliations:** ^1^ Department of Physiology, School of Basic Medical Sciences, Guizhou Medical University, Guiyang, China; ^2^ Key Laboratory of Endemic and Ethnic Diseases, Ministry of Education, Guizhou Medical University, Guiyang, China; ^3^ Key Laboratory of Medical Molecular Biology, Guizhou Medical University, Guiyang, China; ^4^ Department of Pathology, Affiliated Hospital of Guizhou Medical University, Guiyang, China; ^5^ Key Laboratory of Pathogenesis and Drug Research on Common Chronic Diseases, Guizhou Medical University, Guiyang, China

**Keywords:** data analysis, dietary vitamin K intake, human papillomavirus (HPV), HPV-infection, HPV-subtypes

## Abstract

**Objective:** Cervical cancer is a serious potential risk to women’s health, and is closely related to persistent HPV infection. Vitamin K mainly existed in green vegetables, fruit, and dairy products. This research aims to observe the association between vitamin K and HPV-infection.

**Methods:** 13,447 participants from the NHANES were selected. Dietary vitamin K intake was used as the objective independent variable and continuous variable, HPV-infection status was used as the outcome variable, and characteristics of selected participants were used as the covariates.

**Results:** There was a nonlinearity between vitamin K intake and HPV-infection, and the inflection point is 3.81 of log2 vitamin K intake. In a range of 0–3.81, Each one-unit increase in log2 vitamin K intake was associated with a 43% reduction in the risk of HPV infection. When log2 vitamin K intake excess of 3.81, the risk of HPV infection did not continue to decline. The HPV-subtype was not associated with vitamin K intake.

**Conclusion:** There is a nonlinearity between vitamin K intake and HPV-infection status. But HPV-subtype was not associated with vitamin K intake.

## Introduction

Cervical cancer is a serious potential risk to women’s health, and most occurrence remains in almost all low-income countries due to the lack of diagnostic technologies [1]. Cervical carcinogenesis is closely related to persistent HPV infection [4]. Human papillomaviruses (HPVs) are sexually transmitted viruses and are responsible for risk factors for cervical cancer development [2]. To date, more than 200 HPV types have been identified, and approximately 40 types can infect the genital mucosa [3, 4]. According to their oncogenic potential, these HPV types are typically classified into low-risk (LR) and high-risk (HR) HPV types [5]. HR types are the pathogenic factor of cervical cancer, low-risk usually associated with benign genital warts. Specifically, 12 different HR HPV types (HPV16, 18, 31, 33, 35, 39, 45, 51, 52, 56, 58, and 59) were classified as carcinogenic types as there was consistent and sufficient epidemiological and experimental evidence showing their associations with cervical cancer [6, 7]. HPV16 and HPV 18 were the two most prevalent HR HPV types and were responsible for more than 70% of all cervical cancer cases worldwide, while HPV-6 and HPV-11 were most commonly found as LR HPV types [8, 9]. Most HPV infections are naturally eliminated by the immune system of the host within 2 years, about 10% of infections process into persistent infection [2].

The vitamin K family consisted of a 2-methyl-1,4-naphthoquinone ring structure, which is naturally containing vitamin K1 and vitamin K2 [10]. Vitamin K1 is phylloquinone, which is usually present in green vegetables (such as collards, broccoli, and soybean). Vitamin K2 is menaquinone, which is usually present in fermented foods (such as cheese and natto) [11, 12]. Additionally, vitamin K3-K5 are synthetic derivatives [13]. Vitamin K was originally discovered by Henrik Dam in the 1930s, by attention to its vital function of hemostasis [14, 15]. It is reported that vitamin K plays a role as an essential co-factor, which could modify hepatic blood coagulating related proteins after translation [16, 17]. Moreover, vitamin K is related to bone metabolism and angiomatosis [12, 18]. Several research have clarified that vitamin K shows cytotoxicity to cancer cells and suppression of cancer cell proliferation [11, 19]. Most studies pay attention to the balance of redox and oxidative stress in cancer cells dealing with vitamin K [20, 21]. Moreover, other studies indicated that vitamin K is involved in apoptosis through several biochemical pathways [22–26].

Recent studies show that persistent HR HPV-infection is not only a risk factor for cervical cancer, but also includes dietary nutrient intake [27, 28]. Moreover, it is reported that intake of vitamin A, C, D, and E, carotenoids, vegetables, and fruits are the potential prophylaxis to avoid HPV infection [28–31]. However, the relation between vitamin K intake on HPV infection remains unknown. In this study, we used the NHANES data to make a cross-sectional investigation of vitamin K intake on HPV-infection.

## Methods

### Data Source and Ethics Statement

**FIGURE 1:**
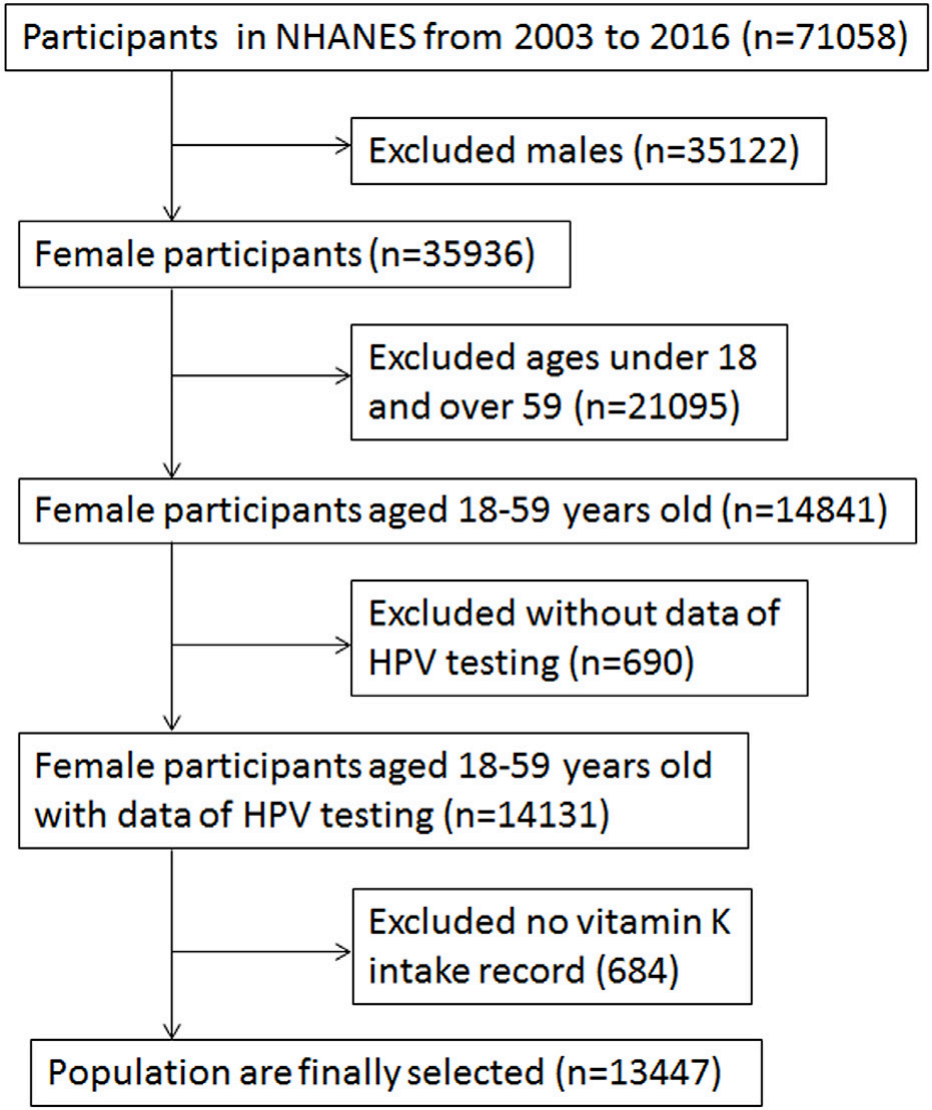
The flow chart is used for data analysis in this study, National Health and Nutrition Examination Survey, the United States, 2003–2016.

All the participants in this analysis were selected from the NHANES, which is a cross-sectional investigation of nutrition and health in the population of the United States of America. It is a continuous public database, with nearly 5,000 people accessed yearly. The participants during 2003–2016 of the NHANES, who meet the analysis criteria were selected (Figure 1). The participants with male, under 18 years old, over 59 years old, without vitamin K intake record, and no HPV testing record was excluded (Figure 1). Finally, 13,447 participants remained for the final data analysis of the risk between vitamin K intake and HPV-infection (Figure 1). More detail of participants are available on the NHANES official website (https://www.cdc.Gov/nchs/nhanes/about_nhanes.htm). The Ethics statement of the observational study was approved by National Center for Health Statistics (NCHS). The Institutional Review Board Approval was available on the official website of the Centers for Disease Control (https://www.cdc.gov/nchs/nhanes/about_nhanes.htm).

### Variables in the Analysis

In this analysis, the independent variable was dietary vitamin K intake. This data is obtained by an average of the dietary vitamin K intake of the participants on the first and second days from the NHANES database. The detail of measurement was available on the NHANES official website (https://wwwn.cdc.gov/nchs/nhanes/ContinuousNhanes/Default.aspx). Moreover, vitamin K intake was also used as the continuous variable in this analysis.

The outcome variable in this analysis was HPV-infection status. The variable of HPV-infection is recorded as a dichotomous variable (1 = infected with HPV; 0 = no infected with HPV). Meanwhile, HPV subtypes were also recorded as a dichotomous variable (1 = high risk subtype; 0 = low risk subtype). HPV infection was tested by HPV DNA genotyping assay, and the detail of the process of HPV testing was available on the NHANES website.

Based on the factors associated with HPV-infection that are reported in the previous study [32–43], we use these factors as the covariates. So these covariates involved in this analysis consist of vitamin K intake data, sociodemographic data, experimental data, questionnaire data, and data from a physical examination. Moreover, the details of these variables are presented in Table 1. The multivariate model used in this analysis is following these covariates.

**TABLE 1:** Characteristics of 13,447 participants, National Health and Nutrition Examination Survey, the United States, 2003–2016.

Vitamin K intake (mcg)	Q1	Q2	Q3	Q4	*p*-value
BMI, mean ± SD, kg/m^2^	29.07 ± 7.57	29.51 ± 7.78	29.44 ± 7.88	28.55 ± 7.63	<0.001
Age, mean ± SD, years	36.09 ± 12.59	36.36 ± 12.33	36.93 ± 12.18	38.07 ± 12.15	<0.001
Frequency of drinking in the past 12 months, mean ± SD, times	3.08 ± 11.90	2.83 ± 5.92	3.84 ± 15.24	3.22 ± 8.11	0.011
Number of sexual partners in the past year, mean ± SD, times	1.50 ± 3.56	1.38 ± 4.31	1.27 ± 1.76	1.36 ± 3.17	0.324
Dietary vitamin B6 intake, mean ± SD, mg	-0.02 ± 1.05	0.49 ± 0.77	0.71 ± 0.74	0.90 ± 0.74	<0.001
Dietary folate intake, mean ± SD, mcg	6.58 ± 0.96	7.17 ± 0.72	7.48 ± 0.68	7.96 ± 0.68	<0.001
Dietary vitamin B12 intake, mean ± SD, mcg	1.17 ± 1.46	1.62 ± 1.21	1.75 ± 1.17	1.81 ± 1.22	<0.001
Dietary vitamin C intake, mean ± SD, mg	4.46 ± 2.30	5.23 ± 1.78	5.70 ± 1.56	6.37 ± 1.30	<0.001
Dietary calcium intake, mean ± SD, mg	9.02 ± 1.08	9.45 ± 0.89	9.60 ± 0.86	9.73 ± 0.79	<0.001
Serum vitamin D level, mean ± SD, ng/mL	58.50 ± 26.89	58.56 ± 26.61	60.02 ± 26.99	62.46 ± 27.83	<0.001
Used contraceptives					<0.001
No	31.28%	29.54%	28.95%	25.73%	
Yes	68.72%	70.46%	71.05%	74.27%	
Used female sex hormone					0.038
No	88.61%	90.21%	288.45%	87.88%	
Yes	11.39%	9.79%	11.55%	12.12%	
Race					<0.001
Mexican American	19.33%	21.99%	20.26%	14.81%	
Other Hispanic	9.34%	8.81%	9.70%	8.00%	
Non-Hispanic White	140.72%	39.01%	38.89%	42.31%	
Non-Hispanic Black	24.55%	23.15%	22.57%	22.00%	
Other races	6.06%	7.05%	8.57%	12.88%	
Education level					<0.001
Less than 9th grade	9.55%	7.85%	200 6.57%	5.16%	
Grades 9–11	17.37%	16.16%	12.39%	9.52%	
High school graduation	24.21%	23.60%	20.74%	16.70%	
College	32.64%	32.18%	34.68%	33.91%	
More than college	16.22%	20.21%	25.61%	34.71%	
Marital status					<0.001
Married or living with partners	52.68%	55.01%	59.78%	59.89%	
Single	47.32%	44.99%	40.22%	40.11%	
Smoking more than 100 cigarettes already					<0.001
No	59.14%	62.41%	65.94%	67.17%	
Yes	40.86%	37.59%	34.06%	32.83%	
Number of vaginal or anal sex in the past year (times)					<0.001
1	4.11%	3.70%	2.96%	2.90%	
2	5.03%	4.15%	2.96%	3.58%	
12–51	26.51%	22.72%	21.68%	22.17%	
52–103	30.40%	33.89%	34.02%	37.46%	
104–364	18.80%	20.29%	22.13%	20.57%	
≥365	13.83%	14.04%	14.70%	12.54%	
0	1.31%	1.22%	1.56%	0.77%	
Poverty income ratio					<0.001
0	32.34%	27.87%	760 24.27%	19.73%	
1	28.33%	25.62%	23.18%	20.49%	
2	13.26%	14.69%	13.12%	13.30%	
3	26.07%	31.82%	39.43%	46.49%	
HPV-infection					<0.001
0	52.39%	55.37%	57.94%	60.45%	
1	47.61%	1500 44.63%	42.06%	39.55%	
HPV subtype					0.024
0	46.55%	770 51.33%	51.20%	48.80%	
1	53.45%	730 48.67%	48.80%	51.20%	

### Statistical Analysis

The objective of this study was to assess the risk of vitamin K intake on HPV-infection. This study was a cross-sectional observation without exploring causality. The clinical characteristics of participants without HPV-infection records were roughly the same as those with the HPV-infection records. So there is no bias in the analysis. Depending on whether continuous variables are normally distributed, the continuous variables are represented as mean (standard deviation, normal distribution) or median (minimum, maximum, skewness distribution). Vitamin K intake was acted as a grouping variable (4 groups: Q1, Q2, Q3, and Q4) to describe the study population. The 4 groups were assessed with One-way ANOVA (normal) or Kruscka Whallis-H (skewed) to evaluate the differences between groups of continuous variables. The differences between groups were evaluated by a rank-sum test. *p* < 0.05 was considered statistically significant.

The data on dietary vitamin intake showed skewed distribution in the participants, so we process vitamin intake with log2 transformation. Results of data analysis were presented in three models: Model 1 (non-adjusted model); Model 2 (adjusted covariables, including Age, Race, Education level, and Marital status); Model 3 (adjusted all the covariables). Adjusted covariates included in Model 3 were screened based on confidence intervals (CI, 95%) and odds ratios (OR), but not based on univariate analysis *p*-values. Since the independent variable in this study is a continuous variable, we conducted the data following sensitivity analysis: 1) the continuous variable was entered into the model to calculate the effect value; 2) the disordered classification variables have entered into the model to observe the effect value; 3) The ordered classified variables were put into the model to calculate the tendency test.

All the covariables were adjusted using a generalized additive model (GAM) to assess the nonlinear relationship, and smooth curves were also plotted. When a nonlinearity was presented, we calculate the inflection point with a recursive algorithm. And then based on both sides of the inflection point, we constructed a piecewise linear model. At last, based on the logarithmic likelihood ratio test *p*-value, the best fitting model was constructed. If *p*-value ≤ 0.05, there was a nonlinearity between vitamin K intake and HPV-infection, otherwise, linearity appeared.

## Results

### Baseline Characteristics of Selected Participants

A total of 13,447 participants in the NHANES from 2003 to 2016 were selected for this analysis. Dietary vitamin K intake was acted as a grouping variable (4 groups: Q1, Q2, Q3, and Q4). Compared with the Q4 group, the participants in other groups (Q1, Q2, and Q3) have higher BMI and are younger. Compared with Q1, participants in other groups (Q1, Q2, and Q3) have more intake of vitamin B6, vitamin B12, vitamin C, folic acid, and dietary calcium. Moreover, no significant differences could be found among different dietary vitamin K intake groups in the number of sexual partners in the last 12 months. But there are significant differences among groups in race, education levels, marital status, smoking over 100, vaginal or anal intercourse times in last year, poverty income ratio, and HPV-infection.

### Unadjusted and Adjusted Models to Analyze Dietary Vitamin K Intake With HPV Infection

To obtain the relationship between vitamin intake and HPV-infection status, three models are processed to investigate the trend of effect value (OR and 95% CI). The OR, 95% CI, and *p*-value in the three models are shown in Table 2. In Model 1 (non-adjusted model), one more unit in log2 dietary vitamin K intake may cause 8% decrease of risk in HPV-infection (0.92 OR, 95% CI: 0.90–0.95). In Model 2 (adjusted covariables, including Age, Race, Education level, and Marital status), one unit more of log2 dietary vitamin intake was related to the 3% decrease in risk of HPV infection (OR: 0.97; 95% CI: 0.94–0.99). However, in Model 3 (adjusted all the covariables), there is no association between log2 dietary vitamin intake and HPV infection (OR: 0.96; 95% CI: 0.89–1.03). Analysis of the HPV subtype shows that the dietary vitamin K intake is not related to the HPV subtype in all models (Table 2).

**TABLE 2:** Linear relation of dietary vitamin K intake and HPV-infection by the weighted binary logistic regression model, National Health and Nutrition Examination Survey, the United States, 2003–2016.

Exposure	Model 1	Model 2	Model 3
	OR, 95% CI, *p*-value	OR, 95% CI, *p*-value	OR, 95% CI, *p*-value
HPV-infection status (negative/positive)			
Vitamin K intake (mcg) (Log 2 transform)	0.92 (0.90, 0.95) <0.0001	0.97 (0.94, 0.99) 0.0200	0.96 (0.89, 1.03) 0.2777
Q1(0–4.94)	1.0	1.0	1.0
Q2 (4.94–5.76)	0.89 (0.81, 0.98) 0.0142	0.95 (0.85, 1.06) 0.3863	0.94 (0.73, 1.20) 0.6264
Q3 (5.77–6.67)	0.80 (0.73, 0.88) <0.0001	0.89 (0.80, 1.00) 0.0480	0.99 (0.76, 1.28) 0.9270
Q4 (6.68–11.99)	0.72 (0.65, 0.79) <0.0001	0.86 (0.77, 0.96) 0.0089	0.80 (0.60, 1.05) 0.1024
P for trend	<0.0001	0.0047	0.1492
HPV subtype (low/risk)			
Vitamin K intake (mcg) (Log 2 transform)	0.97 (0.94, 1.01) 0.1075	0.99 (0.95, 1.03) 0.5805	1.05 (0.96, 1.16) 0.2916
Q1 (0–4.94)	1.0	1.0	1.0
Q2 (4.94–5.76)	0.83 (0.72, 0.95) 0.0079	0.86 (0.74, 1.01) 0.0691	1.13 (0.82, 1.57) 0.4595
Q3 (5.77–6.67)	0.83 (0.72, 0.96) 0.0109	0.84 (0.71, 0.98) 0.0298	0.97 (0.69, 1.38) 0.8751
Q4 6.68–11.99)	0.91 (0.79, 1.06) 0.2264	1.01 (0.85, 1.19) 0.9484	1.33 (0.92, 1.94) 0.1320
P for trend	0.2019	0.8562	0.2550

Outcome variables: HPV-infection status (negative/positive) or HPV subtype (low/risk).

Exposure variables: Vitamin K intake (mcg) (Log 2 transform).

Model 1: no covariates were adjusted.

Model 2: age, race, education level, and marital status was adjusted.

Model 3: all covariates presented in Table 1 were adjusted.

OR: odds ratio.

CI: confidence interval.

### The Nonlinearity of Dietary Vitamin K Intake on HPV Infection and Subtypes

**FIGURE 2:**
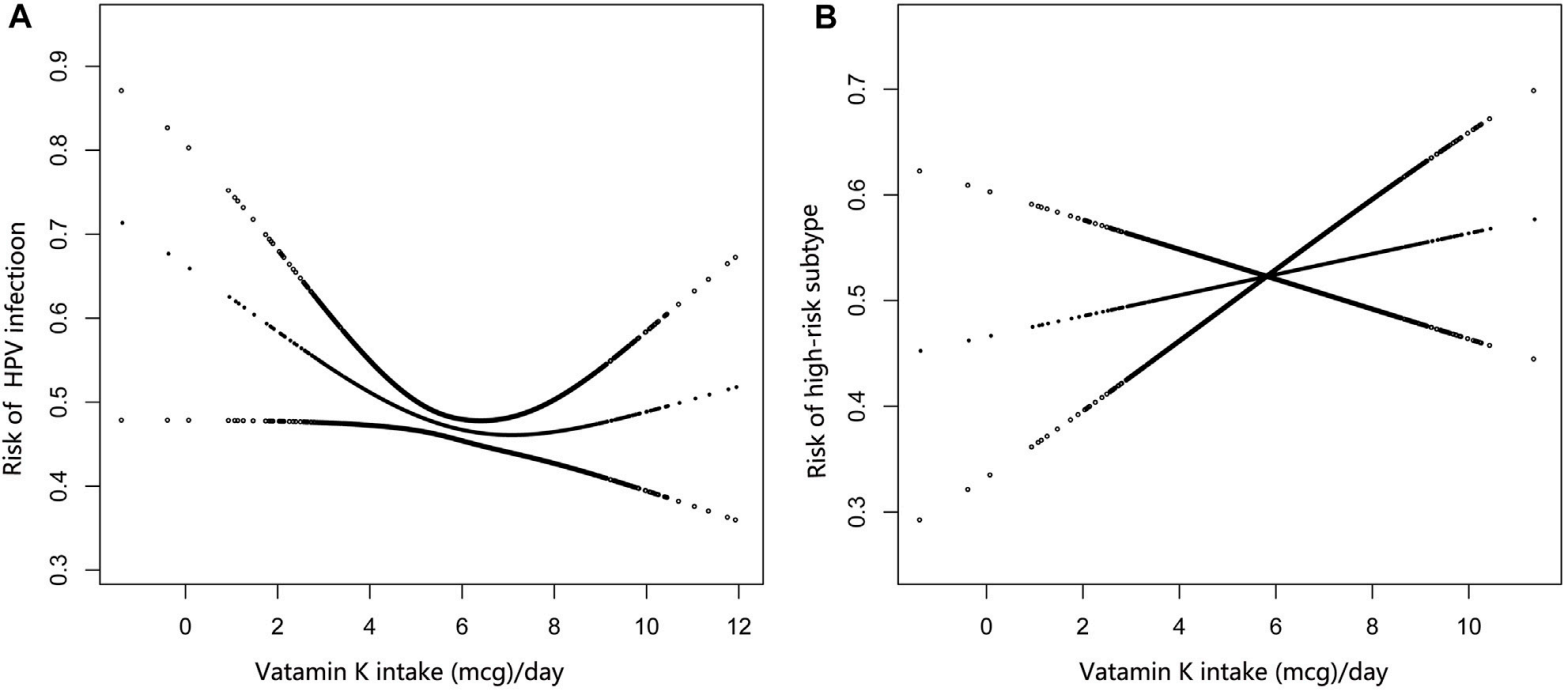
**(A)** A non-linear relationship between dietary vitamin K intake and HPV-infection; **(B)** A non-linear relationship between dietary vitamin K intake and HPV-subtype, National Health and Nutrition Examination Survey, the United States, 2003–2016.

Based on Model 3 (adjusted all the covariables), smooth curve fitting showed a nonlinearity of dietary vitamin K intake with HPV infection (Figure 2A), and the inflection point is 3.81 of log2 vitamin K intake in a recursive algorithm (Table 3). In the range of 0–3.81, each one-unit increase in log2 dietary vitamin K intake was associated with a 43% reduction in the risk of HPV infection (OR: 0.57; 95% CI: 0.37–0.89). However, the risk of HPV infection (OR: 0.99; 95% CI: 0.92–1.07) did not continue to decline with additional increases in dietary vitamin K intake, when log2 dietary vitamin K intake excess 3.81 (Table 3). In addition, the HPV subtype was not associated with dietary vitamin K intake (Figure 2B; Table 3).

**TABLE 3:** Nonlinearity addressing between vitamin K intake and HPV-infection status, National Health and Nutrition Examination Survey, the United States, 2003–2016.

Outcome	HPV infection	HPV subtype
	OR, 95% CI, P	OR, 95% CI, P
Fitting model using the weighted-logistic regression model	0.96 (0.89, 1.03) 0.2687	1.04 (0.94, 1.14) 0.4222
Fitting model using the weighted two-piecewise linear model		
Inflection point	3.81	4.91
≤ inflection point	0.57 (0.37, 0.89) 0.0130	0.96 (0.76, 1.21) 0.7347
> inflection point	0.99 (0.92, 1.07) 0.7497	1.06 (0.95, 1.19) 0.2848
P for the log-likely ratio test	0.011	0.460

The adjusted strategy of covariates was the same as the fully-adjusted model.

OR: odds ratio; CI: confidence interval.

## Discussion

### Dietary Vitamin K Impacts on HPV-Infection

It is reported that persistent HR HPV infection has been considered the risk factor for the development of cervical cancer [44]. However, most HPV infections are naturally eliminated by the immune system of the host, about 10% of infections process into persistent infections [2]. Some lifestyle factors are closely related to HPV-infection, including diet, smoking, sexual behavior, and long-term contraceptive use is closely related to HPV-infected [45]. These lifestyle factors are taken into this analysis and used as variables in the analytical model (Table 1). In other studies, more fruits and vegetable intake is used as prophylaxis to avoid HPV-infection, which are containing an abundance of vitamin A, C, D, and E [29, 30]. However, vitamin K mainly exists in green vegetables, fruits, and dairy products, but the association between vitamin K and HPV-infection is still unclear.

In our study, there is a nonlinearity between dietary vitamin K intake and HPV-infection (Figure 2A). In the range of 0–3.81, each one-unit increase in log2 vitamin K intake was associated with a 43% reduction in the risk of HPV infection. Vitamin K plays a role as an essential co-factor, which could modify hepatic blood coagulating related proteins after translation [16, 17]. Several studies show that vitamin K is believed to have anti-inflammatory properties, mediated by the reduction of circulating inflammatory mediators [46, 47]. The release of pro-inflammatory cytokines is mainly regulated by the NF-kB signaling pathway, and vitamin K is shown to inhibit the release of IkB from NF-kB to allow its entry into the nucleus [48–50]. Human monocyte-derived macrophages showed inhibition of cytokine release (IL-6, TNF-α, IL-1α, IL-1β) when the cells were pretreated with vitamin K2, and the inhibition also showed a dose-response relationship [51–53]. In that case, the association between dietary vitamin K intake (range from 0 to 3.81) and HPV-infection may be likely partially due to the anti-inflammatory action of vitamin K. However, when log2 dietary vitamin K intake is more than 3.81, the risk of HPV infection did not continue to decline with additional increases in vitamin K intake (Table 3), which because of the anti-inflammatory ability of vitamin K may reach the limit. Additionally, the HPV subtype was not associated with vitamin K intake (Figure 2B; Table 3). In a previous study, vitamin K plays an antitumor role in prostate cancer [54]. And vitamin K can stimulate oxidative stress and affect protooncogene expression in carcinoma cells [23]. Persistent HPV-infection leads to chronic inflammations, which may cause mutations in the development of cervical cancer [55]. High intakes of dietary vitamin K may reduce inflammation [46], which may also reduce the risk of cervical cancer.

### Implications for Healthcare Policy

In previous studies, diets containing vitamin B6, B12, C, and folic acid have been reported to prevent from HPV-infection [29, 30], such as cereals, vegetables, fish, fruits, and nuts. The clinical idea of this study is that this is the first time to investigate the association between vitamin K intake and HPV-infection. Our results will offer new understandings and guidance for health policy making of HPV-infection protection. In this study, there is a nonlinearity of dietary vitamin K intake on HPV-infection. In a range of 0–3.81, Each one-unit increase in log2 dietary vitamin K intake was associated with a 43% reduction in the risk of HPV infection. But, once the log2 vitamin K intake excess of 3.81, the risk of HPV infection did not continue to decline. As the result, more than 14.03mcg (2^3.81^ = 14.03) dietary vitamin K intake maybe reduce the risk of HPV-infection. Therefore, our results suggested a guideline of dietary vitamin K intake of more than 14.03 (2^3.81^) mcg.

### Strengths and Limitations of This Study

This analysis was processed with the following strengths. Firstly, the missing data of each covariate is non-randomly missing. So we proceed with dummy variables to adjust them, which could minimize the bias caused by missing variables. This method is usually applied for the observation of a large sample [49]. Secondly, to ensure the stability of data analysis, we used a series of sensitivity analyses to avoid contingency. Thirdly, this study clarified a nonlinear relationship and determined an inflection point, which provided the clinical value of our results. Limits and self-regulated instincts are existing in the human body, so the threshold and saturation effects are very common in most biomedical research. Lastly, we have developed more adequate adjustment strategies in the large sample analysis. For example, folic acid, vitamin B6, B12, and vitamin C associated with HPV infection are recognized as variables [29, 30], which could make the results reliable in the analysis.

However, there are still some limitations in our findings. Firstly, 13,447 participants are women aged 18–59 years from the NHANES database, so the results have restrictions on gender, ethnicity, age, geography, and some important covariables are not contained, such as whether HPV vaccine was injected or not. Secondly, our research is a cross-sectional study. We can not obtain the exact causal relationship between vitamin K intake and HPV-infection. Thirdly, dietary intake was obtained from “24-h dietary recall interviews” of NHANES participants. So, our research has some unavoidable weaknesses such as recall bias, measurement bias, and so on. Finally, in our study, we use regression equations to adjust the possible potential confounders, but there are still some unmeasurable confounders.

### Conclusion

We observed a nonlinearity of vitamin K intake with HPV infection. Each one-unit increase in log2 vitamin K intake is associated with a 43% reduction in the risk of HPV infection. When log2 vitamin K intake excess of 3.81, the risk of HPV infection did not continue to decline with additional increases in vitamin K intake. In addition, the HPV subtype was not associated with vitamin K intake.
